# Intraventricular haemorrhage and posthaemorrhagic ventricular dilatation: moving beyond CSF diversion

**DOI:** 10.1007/s00381-021-05206-8

**Published:** 2021-05-15

**Authors:** Aswin Chari, Conor Mallucci, Andrew Whitelaw, Kristian Aquilina

**Affiliations:** 1 Department of Neurosurgery, Great Ormond Street Hospital, London, UK; 2 Developmental Neurosciences, Great Ormond Street Institute of Child Health, University College London, London, UK; 3 Department of Neurosurgery, Alder Hey Children’s Hospital, Liverpool, UK; 4 Neonatal Neuroscience, Translational Health Sciences, University of Bristol, Bristol, UK

**Keywords:** Germinal matrix haemorrhage, Intraventricular haemorrhage, Posthaemorrhagic ventricular dilatation, Temporising device, Premature, Neuroendoscopic lavage

## Abstract

Advances in medical care have led to more premature babies surviving the neonatal period. In these babies, germinal matrix haemorrhage (GMH), intraventricular haemorrhage (IVH) and posthaemorrhagic ventricular dilatation (PHVD) are the most important determinants of long-term cognitive and developmental outcomes. In this review, we discuss current neurosurgical management of IVH and PHVD, including the importance of early diagnosis of PHVD, thresholds for intervention, options for early management through the use of temporising measures and subsequent definitive CSF diversion. We also discuss treatment options for the evolving paradigm to manage intraventricular blood and its breakdown products. We review the evidence for techniques such as drainage, irrigation, fibrinolytic therapy (DRIFT) and neuroendoscopic lavage in the context of optimising cognitive, neurodevelopmental and quality of life outcomes in these premature infants.

## Introduction

About 1 in 10 babies is born preterm, and this number appears to be increasing in regions with reliable data [1]. The incidence of intraventricular haemorrhage (IVH) in preterm babies remains high at around 25–30% and increases with lower gestational ages and birth weights; the incidence in very premature infants weighing 500–750g is 40% [2, 3]. IVH occurs secondary to germinal matrix haemorrhage (GMH); the germinal matrix is still abundant up to 32 weeks’ gestation and contains fragile and unsupported arterioles and capillaries, with poor capacity to autoregulate. This pressure-passive circulation is susceptible to haemorrhage when rapid changes in blood pressure or respiratory distress occur [4].

Improvements in medical care have increased survival beyond the neonatal period. In these survivors, the presence of IVH is one of the most important determinants of cognitive outcomes and the need for special education [5, 6]. In a cohort of 75 children with large intraparenchymal echodensities, 68% demonstrated cognitive function below 80% of normal [7]. Similar outcomes are identified in larger meta-analyses [8]. Some changes in neonatal care have resulted in effective prevention of IVH. Historically, the incidence of GMH-IVH was highest with the introduction of positive pressure ventilation to neonatal intensive care in the 1960s and subsequently fell as the haemodynamic impact of ventilation was better understood and the use of antenatal steroids became standard care in the 1990s [9, 10]. However, postnatal administration of phenobarbital to control blood pressure fluctuations is associated with no change in IVH or developmental outcomes, and meta-analysis has found little evidence for delayed cord clamping in reducing IVH incidence [11, 12].

Prompt management of IVH and its sequelae is therefore crucial to optimising long-term developmental outcomes in this cohort of premature infants. Brain injury results from the initial GMH itself, the toxic effects of the blood and its breakdown products, particularly free iron in the ventricles, an inflammatory response with influx of activated macrophages and specific injury to oligodendrocyte precursor cells, and the effect of raised intracranial pressure on the developing white matter at a time of rapid brain development [13, 14]. In this review, we outline the current evidence base for management of IVH and its sequelae. In particular, we focus on two aspects—the management of posthaemorrhagic ventricular dilatation (PHVD) and the evolving paradigm of managing the intraventricular blood and its breakdown products in the acute phase.

## Initial diagnosis

Currently, most cases of IVH in premature infants are identified on cranial ultrasound (CrUS), performed soon after birth, routinely or after a period of systemic instability. Neonatal units have established protocols for serial CrUS surveillance following pre-term birth, with closer surveillance at younger gestational ages. The highest risk for bleeding is within the first 48 h of life and most IVH is diagnosed by day 7; surveillance can then be performed less frequently.

**Fig. 1:**
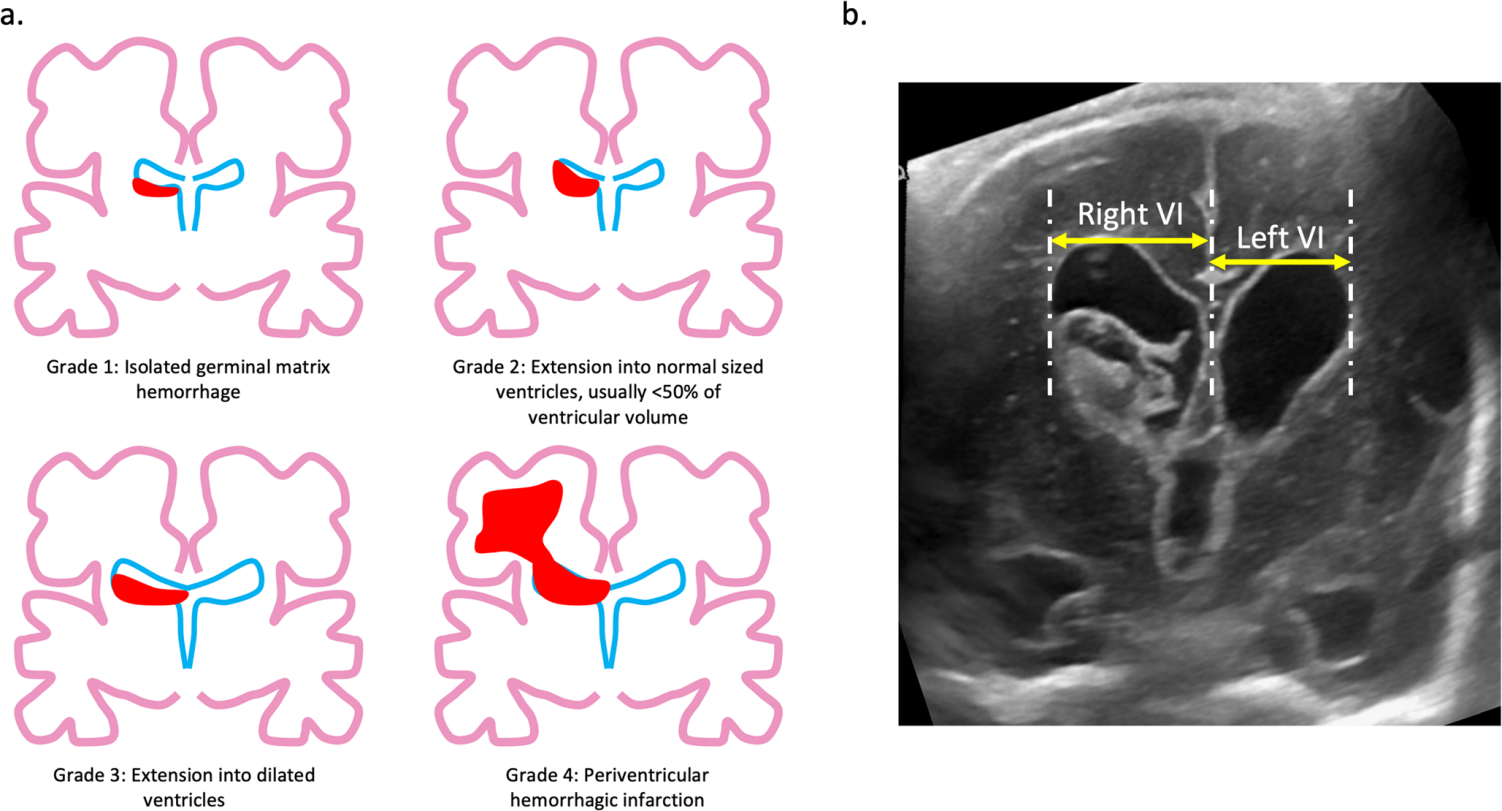
**a** CrUS representations of the different IVH grades. **b** Example measurement of the VI on CrUS

The CrUS is important for establishing two parameters:

- The grade of IVH: this is assessed by the Volpe Grade (Fig. 1a). In general terminology, this is commonly referred to as the synonymous Papile grade, which was initially derived from CT scan findings.
- The degree of PHVD: this is measured by the Ventricular Index (VI, Fig. 1b). The VI is measured from the falx to the lateral wall of the body of the lateral ventricle, measured in the coronal (or axial) plane at the level of the foramen of Monro. Levene produced reference ranges for the VI according to gestational age (weeks 26 to 42). PHVD is a dynamic process and requires serial monitoring. A VI above the 97^th^ centile for the given gestational age is considered high, although specific treatment thresholds are discussed below. Other CrUS measurements such as the anterior horn width (AHW) and thalamo-occipital distance (TOD) are also helpful in guiding management.

Due to the compliance of the neonatal skull and brain, the large extracerebral spaces in preterm infants and the high fluid content of the neonatal brain, VI changes on CrUS precede the clinical signs of ventriculomegaly and raised intracranial pressure (ICP). Tense fontanelle, splayed sutures and rapidly growing head circumference were associated with directly measured mean CSF pressure 9.1 mm Hg ranging up to 34 mm Hg (upper limit of normal neonatal CSF pressure 6 mm Hg) [15]. If left untreated, further signs of progressive ICP rise develop such as bradycardia, desaturation, sun setting and engorged scalp veins.

## Management of PHVD

Traditionally, the management of premature infants with IVH has focussed on the treatment of the ensuing PHVD, which affects about 50% of those with grade 3 or grade 4 IVH. The rationale is that, at least in part, the ventricular dilatation is due to a combination of communicating and obstructive hydrocephalus, and this hydrocephalus is detrimental to the developing brain, resulting in cognitive dysfunction, cerebral palsy and epilepsy. In animal models, the cause of PHVD is multifactorial and involves disruption of normal CSF production, flow and absorption. The accumulation of methaemoglobin from the intraventricular haemoglobin induces the expression of proinflammatory cytokines in a preterm rabbit pup model [16]. Release of transforming growth factor beta from inflammatory cells recruited to the ventricular system and from platelets within the blood clot activates fibroblasts leading to the deposit of fibrin on ependymal surfaces, interfering with the flow and absorption of CSF [17]. In a rat model, the inflammatory response associated with IVH was associated with a significant increase in the secretion of CSF at the choroid plexus epithelium [18].

Definitive treatment of PHVD, in the form of a ventriculoperitoneal (VP) shunt, is associated with high rates of morbidity and failure due to a combination of anaesthetic risk, immunological immaturity, risk of abdominal pathology including necrotising enterocolitis, risk of skin and wound breakdown, valve blockage by blood clot and technical factors associated with operating on such a small neonate. Therefore, the current treatment paradigms involve the use of temporising measures during the neonatal period, with definitive management at or around term-equivalent age. There is currently no role for medical management. A trial of acetazolamide and frusemide in the International PHVD Drug Trial was terminated early as the treatment group had worse outcomes both in terms of shunt placement and death (RR 1.4, p=0.03) as well as neurological disability and death (RR 1.4, p=0.01).

A number of landmark studies have explored the current thresholds for intervention in PHVD. Meta-analysis of trials from the 1980s and 1990s has confirmed that lumbar punctures and ventricular taps do not reduce permanent shunt dependence or neurological disability [19]. Equally, waiting until the PHVD becomes clinically significant may be too late [20]. A retrospective comparative study between Dutch and Canadian cohorts found lower rates of cognitive (p=0.002) and motor (p=0.03) disability in a cohort whose treatment was initiated when the VI exceeded the 97^th^ centile compared to a cohort whose treatment was initiated in the presence of clinical signs of raised ICP [21]. This has recently been evaluated in a randomised controlled trial. The ELVIS (Early versus Late Ventricular Intervention Study) trial has shown that, despite not affecting the composite primary outcome of VP shunt or death, early intervention (VI exceeding the 97^th^ centile or AHW >6mm) is associated with less disability than late intervention (VI exceeding the 97^th^ centile + 4mm or AHW >10mm); the composite rate of death or disability at 24 months’ corrected age was 51% in the late intervention group and 35% in the early intervention group (adjusted OR 0.24, p=0.03) [22, 23].

### Temporising measures

**Fig. 2:**
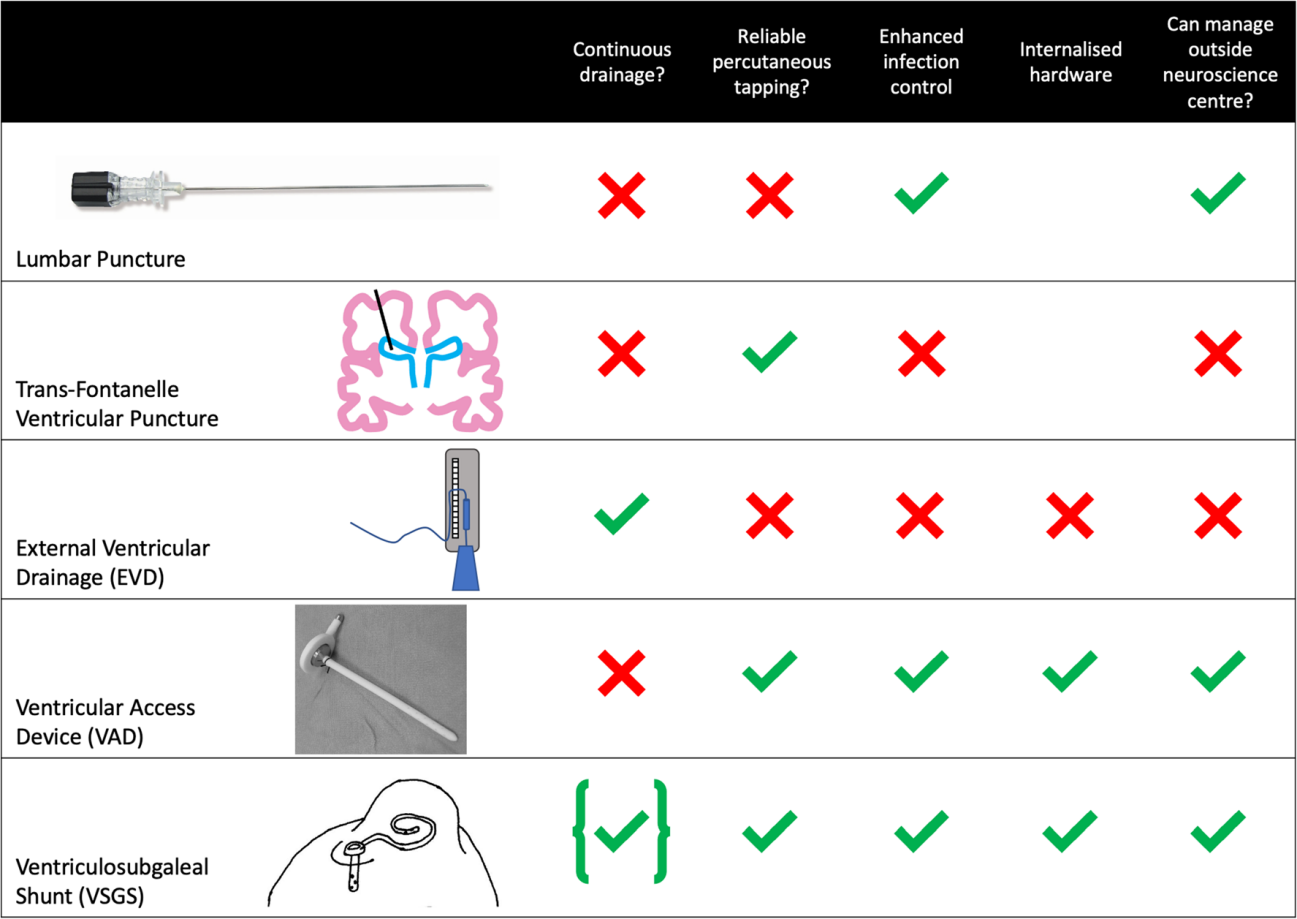
The different temporising measures with accompanying advantages and disadvantages. This image illustrates the relative advantages of the VSG in comparison to other measures, having fully internalised hardware with low infection risk with pseudo-continuous drainage that can be managed remotely outside the neuroscience centre

Once the need for intervention is established, a number of temporising measures may be instituted (Fig. 2). These include lumbar punctures, direct trans-fontanelle ventricular puncture, external ventricular drainage (EVD), ventricular access device (VAD) insertion or ventriculosubgaleal shunt (VSGS) insertion. Lumbar punctures often stop being effective after a number of attempts; ventricular puncture is associated with risks of parenchymal injury, and infection and EVDs require ongoing neurosurgical and neonatal intensive care management, making them unmanageable in many settings.

There is little consensus or standardisation of temporising procedure to perform with significant variation between surgeons and units. This is exemplified in a recent UK survey, which demonstrated a wide variation in practice where respondents noted a preference for VAD (33%), ventricular puncture (25%), VSGS (17%) or repeated lumbar puncture (17%) [24].

Meta-analyses suggest that VAD and VSGS are equivalent in terms of the risks, rates of subsequent need for permanent VP shunt and subsequent shunt revision rates, although earlier treatment is associated with lower rates of VP shunting and neurological disability [25–27]. Following institution of the temporising measures, ongoing monitoring with regular CrUS, head circumference measurement and clinical assessment are important to ensure ongoing control of the PHVD.

#### Permanent CSF diversion

**Fig. 3:**
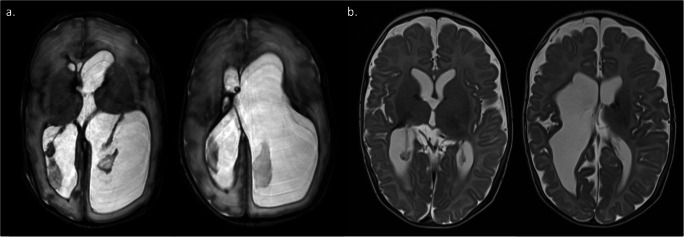
Example MRIs at term-equivalent age with VSG in situ. **a** Ongoing ventricular dilatation. A subsequent VP shunt was inserted to manage this. **b** Well-controlled ventricles. In combination with clinical assessment, VP shunt insertion was deemed not necessary in this infant and, a few weeks later, the VSG was removed

At or around term-equivalent age or when the infant reaches a weight of 1.8–2 kg, there then needs to be a subsequent assessment of whether permanent CSF diversion in the form of a VP shunt is necessary. Individual and institutional practice may vary but this is largely based on the ongoing need for the temporising measures (e.g. VAD taps or VSGS drainage) to control ventricular dimensions [28]. Further information is obtained from clinical assessment, CSF sampling (to ensure protein levels are <1.5g/L and culture negative) and axial magnetic resonance imaging (MRI) (Fig. 3). In addition to the clinical utility, MRI at term-equivalent age, including assessment of the Kidokoro score and ventricular volumes, has been shown to be useful in predicting subsequent neurodevelopmental outcomes [29, 30]. In large series, VP shunt conversion rates in the literature vary widely from 63 to 95% [31–35]. Occasionally in this population, in the context of necrotising enterocolitis, ventricular–atrial shunts may be required when the peritoneum is unable to cope with CSF absorption.

There is little evidence with respect to the optimal valve type to use in this population. Some advocate using low pressure fixed pressure valves to mitigate for the CSF being proteinaceous with the hope of reducing mechanical obstruction. Others advocate programmable valves as the requirement for drainage changes over time; adjusting the pressure is purported to mitigate against potential early (overdrainage) and late (slit ventricles) complications [36]. In terms of catheter choice, the recent BASICS study demonstrated a significant 3-fold reduction in shunt infection using antibiotic impregnated catheters compared to standard or silver impregnated catheters [37].

Beyond the initial haemorrhagic period, the hydrocephalus associated with PHH is traditionally considered as mainly a communicating hydrocephalus and therefore not amenable to treatment with endoscopic third ventriculostomy with choroid plexus coagulation. This has however been used with limited success in some published series [38, 39]. Authors argue that, despite the limited success (37–40%), the demonstrated safety makes it a worthwhile consideration as it may obviate the need for permanent VP shunt, especially if imaging reveals some favourable characteristics, including aqueduct stenosis or a non-scarred and patent prepontine cistern on preoperative MRI.

## Management of the blood and breakdown products

Infants with well-controlled PHVD, even with intervention at an early stage, still suffer from neurological disability [23]. In addition, infants with grades I and II IVH also develop neurological disability [40]. This can be due to the effect of the initial haemorrhage itself (that is only modifiable by preventative strategies to reduce the incidence of GMH) or due to the toxic effects of the blood and blood breakdown products on the developing brain.

The best evidence of our ability to modify this factor comes from the drainage, irrigation and fibrinolytic therapy (DRIFT) studies. DRIFT involved the insertion of two EVDs for drainage, intraventricular administration of recombinant tissue plasminogen activator and irrigation with artificial CSF at a point when the VI was at least on the 97^th^ centile plus 4 mm. The procedure was carried out in the neonatal ICU, and irrigation was continued for at least 72 h to allow clearance of blood and its breakdown products from the CSF. The trial was discontinued prematurely due to the small chance that the short-term primary outcome (requirement for a VP shunt or death) at 6 months will be significantly different between the DRIFT and standard treatment groups. The study however did demonstrate a significant reduction in the proportion of children with severe cognitive disability or death at 2 years in the DRIFT arm, from 71 to 54% (adjusted OR 0.25) [41, 42]. This benefit was maintained at 10-year follow-up [43]. The mean cognitive quotient score was 69.3 in the DRIFT group and 53.7 in the standard treatment group; this improved cognition at 10 years was equivalent to a 2-year developmental delay [43]. These results represented the most marked improvement in cognition and survival with any intervention after IVH and PHVD. A study that used network meta-analysis methodology to evaluate the outcome of ten different trials for PHVD also demonstrated that DRIFT is the most efficacious and the most likely treatment to improve outcomes [44]. Despite this, however, the DRIFT technique has not been widely adopted and has not changed standard practice due to the resource-intensive nature of the treatment and the potential risks of secondary haemorrhage.

**Fig. 4:**
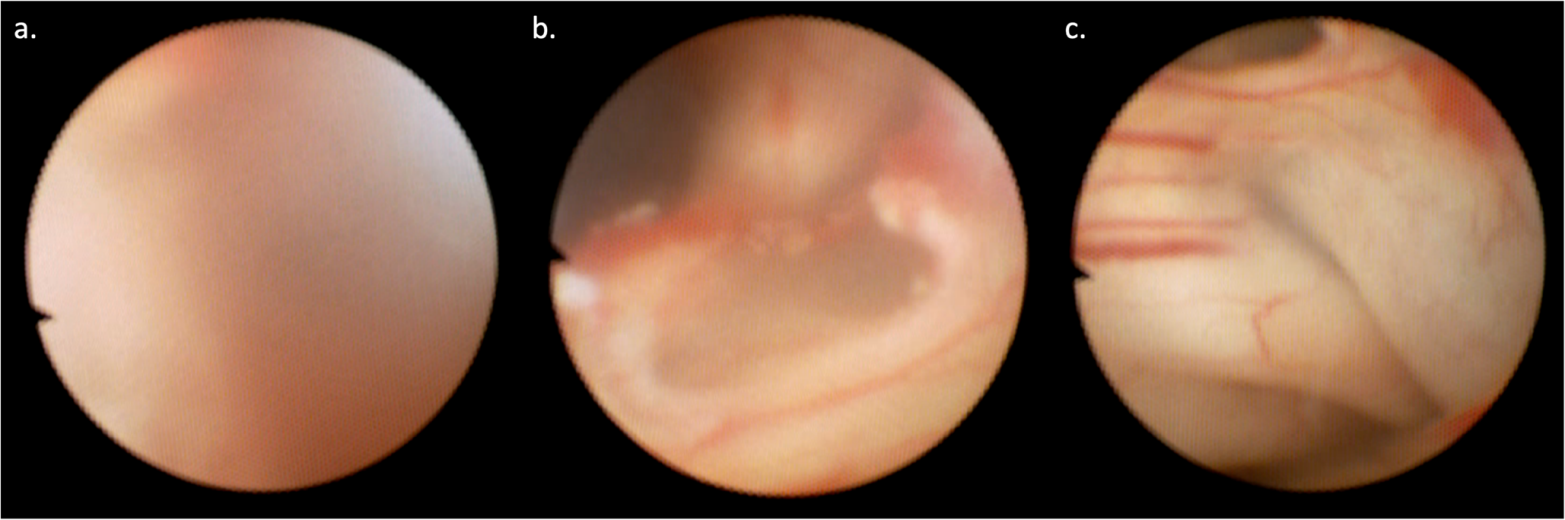
Typical views during the **a** beginning, **b** middle and **c** end of a neuroendoscopic lavage procedure. Whilst the CSF is bloodstained and views are very difficult to obtain at the beginning of the procedure, as the procedure progresses, the CSF becomes clearer, affording better views of the ventricular walls and anatomy. Towards the end, the foramina of Monro are visualised and a septostomy is attempted to ensure CSF flow (not shown)

An alternative to DRIFT that is less resource intensive is neuroendoscopic lavage (Fig. 4), which involves the use of an endoscope to gently irrigate the ventricular CSF, clearing the ventricles of any large clots and restoring CSF flow via a septostomy and ensuring patency of the foramina of Monro. To date, there is no class I evidence of its efficacy but retrospective evaluations have had encouraging results with the procedure showing safety, reductions in VP shunt insertion and revision rates and favourable neurodevelopmental outcome [45–49]. There have been no prospective comparative trials. Specifically, the neurodevelopmental outcomes have not been systematically assessed; the only study to do so only assessed cognitive outcomes in 61% of infants alive at 2 years, with 30% having cognitive profiles within the normal range [45].

## Future directions

There are multiple avenues to improve the long-term outcomes of premature infants with IVH.

A first important consideration is the selection and measurement of endpoints in clinical studies. Whilst early trials focussed on VP shunt dependence and revision rates, results from the DRIFT and ELVIS studies have shown that developmental outcomes can improve despite unchanged VP shunt dependency. Patient involvement initiatives have confirmed that cognitive and developmental outcomes are the most important outcomes for patients, and this should be the focus of future trials [50, 51]. This may be further strengthened and homogenised by the development of core sets of outcome measures that will facilitate cross-study comparisons and the progress of international registries such as TROPHY [52, 53]. Whilst the current standard in these studies has been developmental assessment at 2 years’ corrected gestational age, further work may identify earlier biomarkers of eventual neurodevelopmental outcome that will facilitate more rapid investigation [54].

The prevention of GMH, IVH and PHVD is crucial to improving neurological outcomes. Although detailed discussion of this may be beyond the scope of this review, risk factors including fetal, maternal, delivery-related and haemodynamic have been reviewed elsewhere and it remains to be seen whether these can effectively be modified to alter incidence [9].

In terms of managing the PHVD, recent progress has advocated for early intervention via temporising measures, and it seems that VAD and VSGS are largely equivalent in efficacy and long-term outcomes. The impact of adjustable versus fixed pressure valves for definitive VP shunts is an area that requires further study, as is the role of non-shunt procedures such as endoscopic third ventriculostomy with choroid plexus coagulation, especially in resource-limited settings.

Further research into the direct management of intraventricular blood and its breakdown products is required. Despite the incidence and developmental impact of IVH and PHVD, only about 700 infants have been enrolled into intervention trials, and fewer than 500 have had long-term developmental measures reported [44]. The role of surgical and medical therapies in removing the blood and reducing its toxicity on the developing brain requires robust evaluation and may hold the potential to further improve developmental outcomes in premature neonates with IVH.

## Conclusion

The management of GMH-IVH and PHVD in premature infants has evolved considerably over the last few decades. The management of PHVD at an early stage through temporising devices followed by subsequent permanent CSF diversion has led to reductions in morbidity and complications. The paradigm of clearing the blood and its breakdown products is in its relative infancy, with neuroendoscopic lavage showing promise of being a safe and effective procedure. In conjunction with prevention of IVH, this holds the key to progress in optimising cognitive, neurodevelopmental and quality of life outcomes in these premature infants.

## Data Availability

Not applicable
